# TLR4-Upregulated IL-1β and IL-1RI Promote Alveolar Macrophage Pyroptosis and Lung Inflammation through an Autocrine Mechanism

**DOI:** 10.1038/srep31663

**Published:** 2016-08-16

**Authors:** Xingying He, Yongbing Qian, Zhigang Li, Erica K. Fan, Yuehua Li, Liang Wu, Timothy R. Billiar, Mark A. Wilson, Xueyin Shi, Jie Fan

**Affiliations:** 1 Department of Anesthesiology, Changzheng Hospital, Second Military Medical University, Shanghai 200003, China; 2 Department of Surgery, University of Pittsburgh School of Medicine, Pittsburgh, PA 15213, USA; 3 Department of Critical Care Medicine, Shanghai First Hospital, Jiaotong University School of Medicine, Shanghai 201600, China; 4 Department of Biological Sciences, University of Pittsburgh School of Arts and Science, Pittsburgh, PA 15213, USA; 5 Research and Development, Veterans Affairs Pittsburgh Healthcare System, Pittsburgh PA 15240, USA; 6 McGowan Institute for Regenerative Medicine, University of Pittsburgh, Pittsburgh, PA 15219, USA; 7 Department of Anesthesiology, Shanghai Xinghua Hospital, Jiaotong University School of Medicine, Shanghai 200092, China

## Abstract

Acute lung injury (ALI) is a major component of multiple organ dysfunction syndrome (MODS) following pulmonary infection. Alveolar macrophages (AM) are at the center of the pathogenesis of the development of ALI. Interleukin-1β (IL-1β) is one of the key pro-inflammatory mediators, and its maturation is tightly controlled by the formation and activation of the inflammasome. The biological effects of IL-1β are mediated through IL-1 receptor (IL-1R). In this study, we investigated the influence of LPS-induced IL-1β release and IL-1RI upregulation on the development of lung inflammation. We demonstrated that in AM, LPS-TLR4 signaling not only activates Nlrp3 inflammasome activation and subsequent release of IL-1β, but also up-regulates IL-1RI expression on AM surface through MyD88 and NF-κB dependent signaling. The upregulated IL-1RI, therefore, sensitizes AM to IL-1β and results in pyroptosome formation, which in turn leads to AM pyroptosis, a type of caspase-1-dependent inflammatory cell death. We further showed that AM pyroptosis exaggerates lung inflammation. The present study demonstrates a novel mechanism underlying LPS-induced innate immunity; that is, a secondary upregulation of IL-1β-IL-1RI signaling is responsible for AM pyroptosis and augmented lung injury in response to LPS.

The development of acute lung injury (ALI) and acute respiratory distress syndrome (ARDS) in individuals following pulmonary infection contributes significantly to morbidity and mortality in this patient population^1^. Alveolar macrophages (AM) play a central role in the development of ALI following infection through synthesis and release of a number of inflammatory mediators^2^,^3^. Interleukin-1β (IL-1β) is one of the key pro-inflammatory mediators of inflammation. IL-1β not only causes inflammation itself, but more importantly also induces the expression of many other pro-inflammatory cytokines and adhesion molecules, which further exaggerate inflammation^4^,^5^.

The production of active IL-1β is tightly controlled by the formation and activation of the inflammasome, which is comprised of NOD-like receptors (NLRs), apoptosis-associated speck-like protein containing a CARD domain (ASC), and caspase-1 4,6,7. Caspase-1 is synthesized as an inactive 45-kDa protein (pro-caspase-1) that undergoes autocatalytic processing after assembly of the inflammasome in response to an appropriate stimulus^8^. IL-1β is synthesized initially as an inactive precursor molecule (pro- IL-1β p35), which must be cleaved by caspase-1 at amino acid position 116 to produce the actively mature IL-1β (p17) that is then secreted in response to stimulating signals.

The biological effects of IL-1β are mediated through the IL-1 receptor I (IL-1RI). IL-1RI belongs to IL-1R subfamily which is characterized by the presence of extracellular immunoglobulin-like (Ig) domains and an intracellular TIR domain. IL-1RI and IL-1R accessory protein (IL-1RAcP) forms a receptor complex specifically sensing IL-1 family members including IL-1α, IL-1β, and IL-1 receptor antagonist (IL-1Ra)^9^. A well accepted signaling pathway following IL-1RI activation is mediated through adaptors MyD88-IRAK-1-Tollip^10^,^11^. These adaptors bridge the IL-1RI receptors to the intracellular molecules that transduce their signals into a biological response and play a central role in innate immunity^12^.

Pyroptosis was first identified in the macrophage, which presented rapid lysis after infection with Shigella flexneri^13^. Pyroptosis is an inflammatory form of cell death, typically triggered by inflammasome formation, which leads to caspase-1 activation^14^. An early step in pyroptosis is the formation of small cation-permeable pores in the plasma membrane. This process is caspase-1 dependent and dissipates cellular ionic gradients, producing a net increased osmotic pressure, water influx, cell swelling and, eventually, osmotic lysis and release of inflammatory intracellular contents^15^,^16^,^17^. These features, along with the unique biochemical requirement for caspase-1 and release of proinflammatory intracellular contents, distinguish pyroptosis from other cell death programs such as apoptosis and necroptosis^18^. We have previously reported a mechanism underlying endogenous danger signaling-induced macrophage pyroptosis, in which HMGB1-activated pyroptosome, a supramolecular assembly of ASC dimers, plays a critical role. However, AM pyroptosis occurs in a setting of LPS-induced lung inflammation and its underlying mechanism remains unclear.

In this study, we investigated the role of LPS-induced IL-1β maturation and IL-1RI upregulation in promoting AM pyroptosis and lung inflammation. We demonstrated that LPS acting through TLR4 signaling and NF-κB pathway upregulates IL-1RI in AM. Meanwhile, LPS-TLR4 signaling activates the Nlrp3 inflammasome, which in turn, promotes IL-1β maturation and release. We further showed that the LPS-induced IL-1β secretion through an autocrine mechanism activates the upregulated IL-1RI, and thus, promotes AM pyroptosis, which subsequently enhances lung inflammation.

## Results

### LPS upregulates IL-1RI cell surface expression in AM

**Figure 1:**
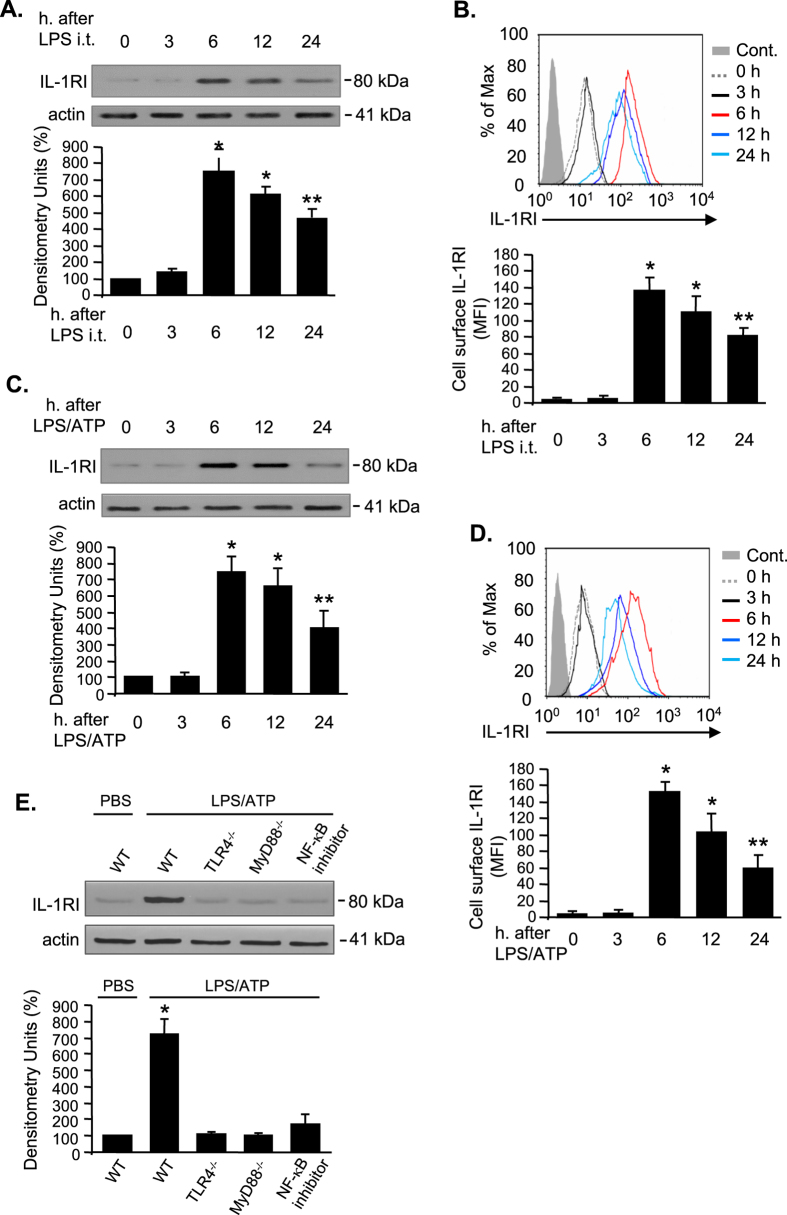
LPS upregulates IL-1RI cell surface expression in alveolar macrophages. (**A**,**B**) WT (C57BL/6) mice were intratracheally injected with LPS (1 mg/kg B.W. in 0.2 ml of saline) using Micro Sprayer Syringe. At 0–24 h after LPS injection, AM were collected from BAL fluid, and Western blot was performed to measure IL-1RI expression in the AM, and the densitometry units of the IL-1RI bands were analyzed (**A**). The AM were also stained with PE-tagged anti-IL-1RI antibody for analysis of cell surface expression of IL-1RI (**B**). (**C**,**D**) AM isolated from normal WT mice were incubated with LPS (1 μg/ml) and ATP (5 mM) for 0–24 h. IL-1RI expression in the AM was then measured by Western blot (**C**) and IL-1RI cell surface expression was analyzed by flow cytometry (**D**). (**E**) AM isolated from WT, TLR4^−/−^, and MyD88^−/−^ mice were treated with LPS and ATP for 6 h; and in some WT AM, NF-κB inhibitor SN50 (200 μg/ml) was added 30 min before LPS/ATP treatment, and IL-1RI expression in the AM was then measured by Western blot. All images are representatives of five independent experiments, and the samples were run on the same gel. The graphs depict the value of mean and SEM. *p < 0.05 compared with the groups labeled with no asterisk, **p < 0.05 compared with the groups labeled without or different type of asterisk.

To study the effect of LPS on IL-1RI expression, AM were collected from C57BL/6 (Wild type, WT) mice at 0, 3, 6, 12, 24 h after intratracheal injection (i.t.) of LPS. As shown in Fig. 1A, the expression of IL-1RI increased and reached a peak at 6 h after LPS i.t., and then trended to decrease during the period of 12 to 24 h after LPS treatment. Since IL-1RI on cell surface is the only form that is physiologically responsible for IL-1β recognition, we thus measured the cell surface expression of IL-1RI in AM at 0–24 h after LPS i.t. using flow cytometry. Consistent with the changes in total IL-1RI protein expression, the cell surface expression of IL-1RI increased most noticeably at 6 h after LPS treatment and lasted for 24 h after the treatment (Fig. 1B). To further verify the effect of LPS on IL-1RI expression, AM were isolated from WT (C57BL/6) mice and treated with LPS and ATP for 0–24 h *in vitro*. We found that both total and cell surface expression of IL-1RI increased and reached a peak at 6 h after LPS/ATP treatment and lasted for 24 h (Fig. 1C–D), which is similar to observation from *in vivo* studies. Furthermore, to determine the receptor signaling pathway that mediates IL-1RI up-regulation, AM isolated from WT, TLR4^−/−^, and MyD88^−/−^ mice were treated with LPS for 6 h; and in some cases, NF-κB inhibitor SN50 (Enzo Life Sciences, Inc. Farmingdale, NY) was added to WT AM 30 min before LPS treatment. The results demonstrated that TLR4 and MyD88 deficiency as well as NK-κB inhibition completely prevented the LPS-induced up-regulation of IL-1RI, indicating the role of TLR4, MyD88, and NF-κB in inducing IL-1RI expression in AM (Fig. 1E).

### LPS induces IL-1β maturation and release through Nlrp3 inflammasome

**Figure 2:**
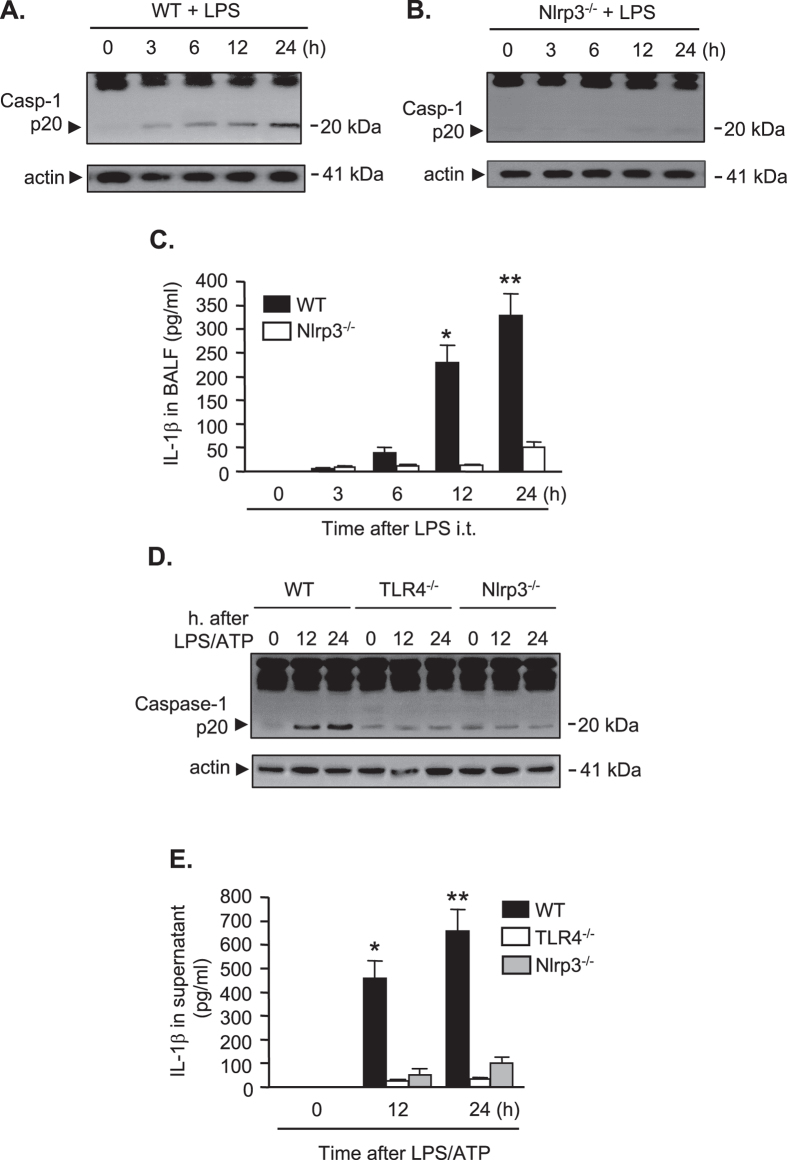
LPS induces IL-1β maturation and release through Nlrp3 inflammasome. (**A–C**) WT (C57BL/6) mice and Nlrp3^−/−^ mice were intratracheally injected with LPS (1 mg/kg B.W. in 0.2 ml of saline) using Micro Sprayer Syringe. BAL was performed at 0–24 h after LPS injection and AM were isolated from the BAL fluid. Caspase-1 cleavage fragment p20 in the AM was detected by Western blot (**A**,**B**), and IL-1β concentration in the BAL fluid was quantified by ELISA (**C**). (**D**,**E**) AM isolated from WT, TLR4^−/−^, and Nlrp3^−/−^ mice were treated with LPS (1 μg/ml) and ATP (5 mM) for 1, 12, and 24 h. Caspase-1 cleavage fragment p20 in the AM was detected by Western blot (**D**), and IL-1β concentration in the cell culture media was quantified by ELISA (**E**). All images are representatives of five independent experiments, and the samples were run on the same gel. The graphs depict the value of mean and SEM, n = 5. *p < 0.05 compared with the groups labeled with no asterisk, **p < 0.05 compared with the groups labeled without or different type of asterisk.

Next, we determined the changes in caspase-1 activation and IL-1β maturation in AM after *in vivo* LPS i.t. AM were collected from WT mice treated with LPS i.t. for 0, 3, 6, 12, and 24 h, and caspase-1 cleavage in the AM was detected. The expression of caspase-1 cleavage product p20 fragment markedly increased during a period between 6 h and 24 h after LPS i.t. (Fig. 2A). Our previous studies have suggested that the Nlrp3 inflammasome is a essentially a key machine for the maturation of caspase-1 and IL-1β in the mouse macrophage following TLR4 activation^19^. To address whether Nlrp3 is required for LPS-induced caspase-1 activation, Nlrp3^−/−^ mice were treated with LPS i.t., and at 0–24 h after LPS, AM were collected for detecting caspase-1 p-20 fragments by Western blotting. As shown in Fig. 2B, LPS failed to induce caspase-1 activation at all time points in Nlrp3^−/−^ AM, indicating an essential role for Nlrp3 in mediating LPS-induced caspase-1 activation.

Furthermore, we compared the changes of IL-1β in bronchoalveolar lavage fluid (BALF) after LPS i.t. between WT and Nlrp^−/−^ mice. Figure 2C shows that LPS markedly increased IL-1β level in the BALF from the WT mice in a time-dependent manner, and this effect of LPS was largely aborted in Nlrp3^−/−^ mice. These *in vivo* observations were also observed *in vitro*. AM were collected from WT, TLR4^−/−^, and Nlrp3^−/−^ mice, and then treated with LPS and ATP for 12 and 24 h *in vitro*. LPS/ATP significantly increased caspase-1 cleavage and IL-1β release in the WT AM at both time points; however, these effects of LPS were greatly diminished in the AM isolated from TLR4^−/−^ or NLRP3^−/−^ mice (Fig. 2D,E).

Collectively, these results indicate that LPS induces IL-1β maturation and release through the Nlrp3 inflammasome.

### IL-1β-IL-1RI signal mediates LPS-induced pyroptosome formation

**Figure 3:**
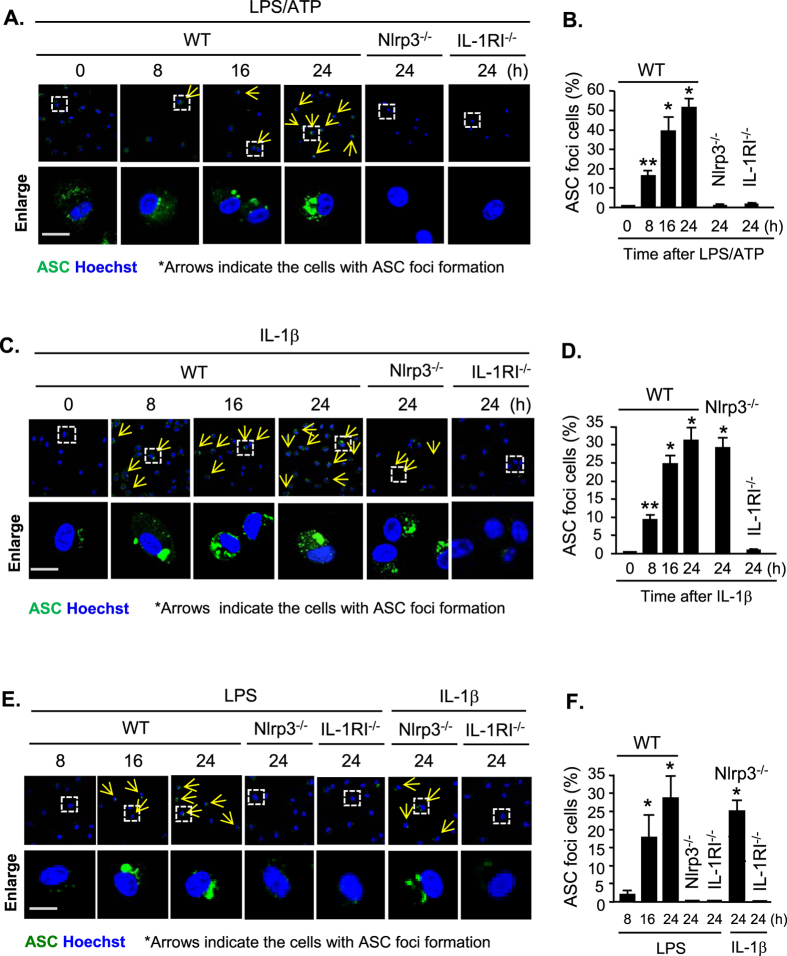
IL-1β-IL-1RI signal mediates LPS-induced pyroptosome formation. (**A**,**B**) Confocal microscopy of immunofluorescence of ASC foci in AM. AM isolated from WT, Nlrp3^−/−^, and IL-1RI^−/−^ mice were treated with LPS (1 μg/ml) and ATP (5 mM) for 0–24 h, and then ASC (green) were detected by immunofluorescence and confocal microscopy. Original magnification × 600, scale bar, 10 μm. (**C**,**D**) Confocal microscopy of immunofluorescence of ASC foci in AM. AM isolated from WT, Nlrp3^−/−^, and IL-1RI^−/−^ mice were treated with IL-1β (5 ng/ml) for 0–24 h, and ASC (green) were then detected by immunofluorescence and confocal microscopy. Original magnification × 600, scale bar, 10 μm. (**E**,**F**) WT, Nlrp3^−/−^, and IL-1RI^−/−^ mice were intratracheally injected with LPS (1 mg/kg B.W. in 0.2 ml of saline) or IL-1β (5 μg/kg B.W. in 0.2 ml of saline) using Micro Sprayer Syringe. BAL was performed at 8–24 h after the treatment and AM were isolated from the BAL fluid. ASC (green) were detected by immunofluorescence and confocal microscopy. Original magnification × 600, scale bar, 10 μm. All images are representatives of five independent experiments, and graphs depict the value of mean and SEM, n = 5. *p < 0.05 compared with the groups labeled without or different type of asterisk, **p < 0.05 compared with the groups labeled without or different type of asterisk.

The formation of the ASC pyroptosome is a unique character for caspase-1 induced pyroptosis^20^. To detect the ASC pyroptosome formation, WT AM were treated with LPS and ATP *in vitro* for 0–24 h, and ASC was visualized by florescence-tagged ASC antibody and confocal microscopy. We found that starting from 8 h after LPS/ATP treatment, ASC foci formed in WT AM cytoplasm (Fig. 3A,B). By contrast, ASC foci were not observed in the AM isolated from Nlrp3^−/−^ and IL-1RI^−/−^ mice following LPS/ATP treatment for up to 24 h (Fig. 3A,B). Direct treatment of AM with IL-1β induced ASC pyroptosome formation in the WT and Nlrp3^−/−^ AM, but not in IL-1RI^−/−^ mice, indicating that IL-1β acting through IL-1RI mediates LPS-induced ASC pyroptosome formation in AM (Fig. 3C,D). The *in vitro* results were then verified *in vivo* by administering WT, Nlrp3^−/−^, and IL-1RI^−/−^ mice with LPS i.t. As shown in Fig. 3E,F, LPS i.t. induced ASC foci formation in the AM from WT mice but not in that from Nlrp3^−/−^ and IL-1RI^−/−^ mice. However, IL-1β i.t. was able to induce ASC pyroptosome formation in the AM from WT and Nlrp^−/−^ mice, but not in the AM from IL-1RI^−/−^ mice (Fig. 3E,F).

These results indicate that the formation of ASC pyroptosome in response to LPS is a secondary event dependent upon Nlrp3 inflammasome activation, IL-1β release, and IL-1β-IL1RI signaling.

### The IL-1β-IL-1RI signal mediates LPS-induced AM pyroptosis

**Figure 4:**
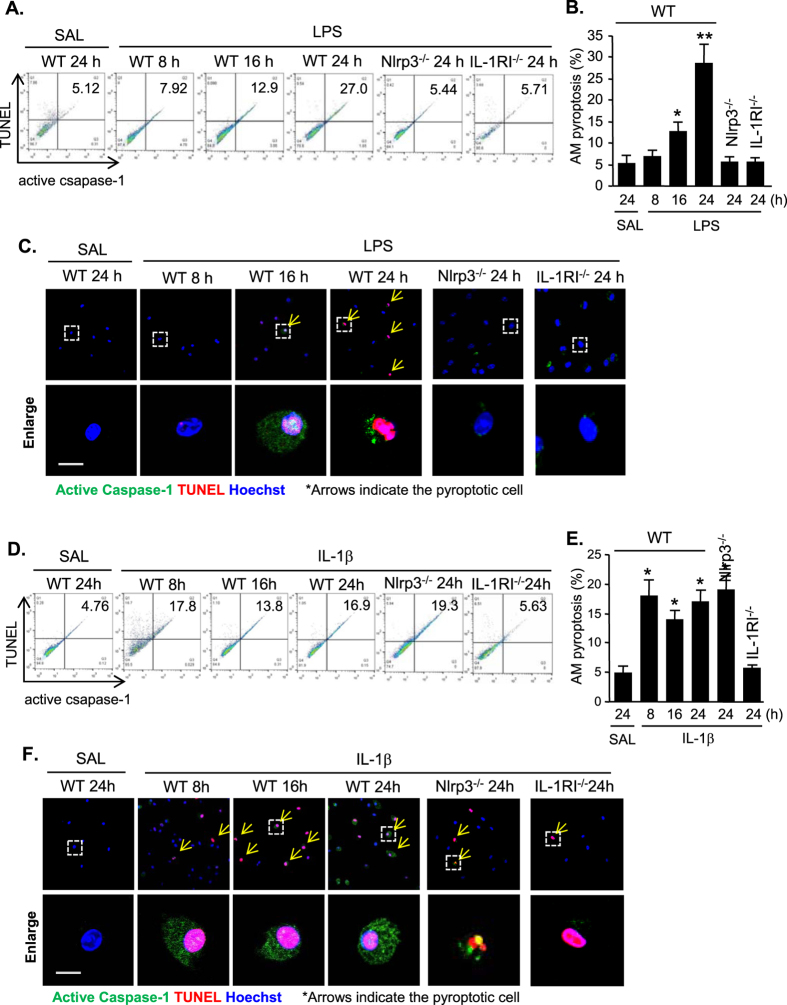
IL-1β-IL-1RI signal mediates LPS-induced AM pyroptosis. (**A–C**) WT (C57BL/6) mice and Nlrp3^−/−^ mice were intratracheally injected with LPS (1 mg/kg B.W. in 0.2 ml of saline) using a Micro Sprayer Syringe. BAL was performed at 0–24 h after LPS injection and AM were isolated from the BAL fluid. DNA fragmentation and caspase-1 activation in the AM were detected by labeling the cells with TUNEL and Alexa Fluor 488 labeled caspase-1 FLICA, respectively, and quantified by flowcytometry (**A**,**B**) and confocal microscopy (**C**). (**D–F**) WT (C57BL/6) mice and Nlrp3^−/−^ mice were intratracheally injected with IL-1β (5 μg/kg B.W. in 0.2 ml of saline) using a Micro Sprayer Syringe. BAL was performed at 0–24 h after LPS injection and AM were isolated from the BAL fluid. DNA fragmentation and caspase-1 activation in the AM were detected by labeling the cells with TUNEL and Alexa Fluor 488 labeled caspase-1 FLICA, respectively, and quantified by flowcytometry (**D**, **E**) and confocal microscopy (**F**). Original magnification for confocal images is × 600, scale bar, 10 μm. All images are representatives of five independent experiments, and graphs depict the value of mean and SEM, n = 5. *p < 0.05 compared with the groups labeled without or different type of asterisk, **p < 0.05 compared with the groups labeled without or different type of asterisk.

Pyroptosis is a caspase-1-dependent regulated cell death^17^. To determine if pyroptosome formation and caspase-1 activation are followed by AM pyroptosis, we treated WT mice, Nlrp3^−/−^ mice and IL-1RI^−/−^ mice with LPS i.t., and at 8–24 h after the treatment, AM were collected from the mice. DNA fragmentation and caspase-1 activation in the AM were detected by labeling the cells with TUNEL and Alexa Fluor 488 labeled caspase-1 FLICA, respectively, and quantified by flowcytometry and confocal microscopy. As shown in Fig. 4A–C, starting at 16 h, LPS induced an increase in AM pyroptosis, which was further increased at 24 h after LPS treatment. Nlrp3 or IL-1RI deficiency, however, prevented the LPS-induced AM pyroptosis (Fig. 4A–C). Furthermore, we directly treated WT mice, Nlrp3^−/−^ mice and IL-1RI^−/−^ mice with IL-1β i.t. and measured the changes in AM pyroptosis. Flowcytometry and confocal microscopy demonstrated that beginning at 8 h after IL-1β i.t., AM pyroptosis increased in WT and Nlrp3^−/−^ mice, but not in IL-1RI^−/−^ mice, indicating an important role of IL-1β in mediating LPS-induced AM pyroptosis (Fig. 4D–F).

### The influence of AM pyroptosis on lung inflammation

**Figure 5:**
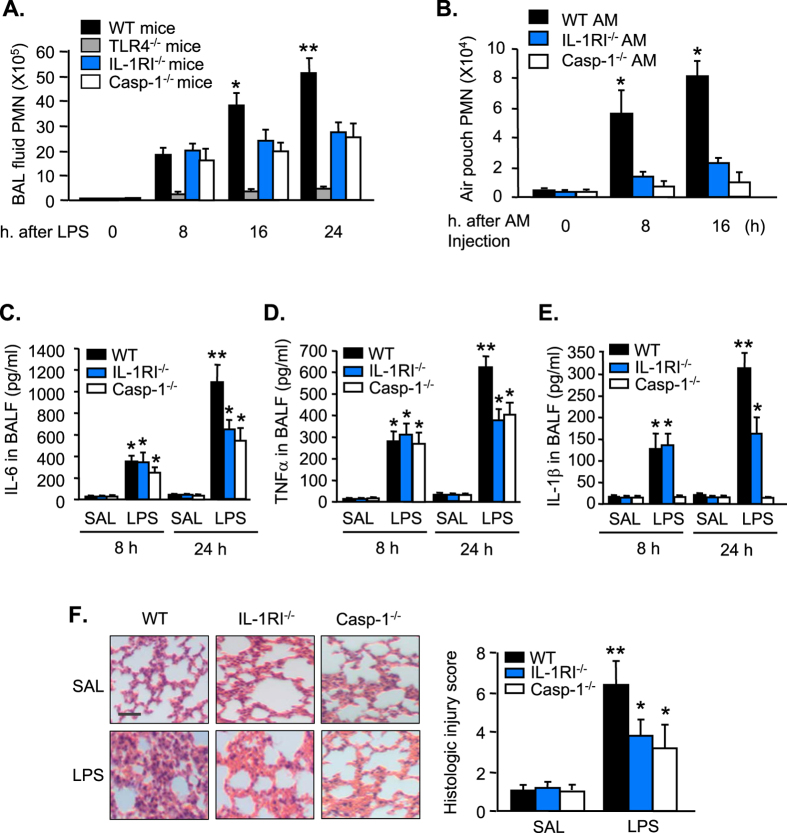
The influence of AM pyroptosis on lung inflammation. (**A**) WT, TLR4^−/−^, IL-1RI^−/−^, and caspase-1 (Casp-1)^−/−^ mice were intratracheally injected with LPS (1 mg/kg B.W. in 0.2 ml of saline) using a Micro Sprayer Syringe. BAL was performed at 0–24 h after LPS injection and PMN in the BAL fluid were counted. (**B**) AM were isolated from WT, IL-1RI^−/−^, and caspase-1 (Casp-1)^−/−^ mice and treated with LPS/ATP *in vitro* for 24 h for induction of pyroptosis. The AM (1 × 10^5^ cells/ml saline/mouse) were then injected into the air pouch of WT mice. At 0, 8, and 16 h after macrophage injection, air pouch lavage fluid was collected for PMN counts. (**C**–**E**) WT, IL-1RI^−/−^, and caspase-1 (Casp-1)^−/−^ mice were intratracheally injected with LPS (1 mg/kg B.W. in 0.2 ml of saline) or saline (SAL, 0.2 ml) using Micro Sprayer Syringe. BAL was performed at 8 and 24 h after LPS injection and IL-6, TNFα, and IL-1β in the BAL fluid were measured by ELISA. (**F**) WT, IL-1RI^−/−^, and caspase-1 (Casp-1)^−/−^ mice were intratracheally injected with LPS (1 mg/kg B.W. in 0.2 ml of saline) or saline (SAL, 0.2 ml) using a Micro Sprayer Syringe. At 24 h after LPS i.t., lung histology was assessed with H&E staining (original magnification × 400, scale bar, 100 μm) and scored. All images are representatives of five independent experiments, and graphs depict the value of mean and SEM, n = 5. *p < 0.05 compared with the groups labeled with no asterisk, **p < 0.05 compared with the groups labeled without or different type of asterisk.

To determine the impact of AM pyroptosis on lung inflammation, we measured PMN infiltration in the lungs at 0 to 24 h after LPS i.t. by counting PMN in BAL fluid. As shown in Fig. 5A, LPS induced in WT mice a gradual increase in PMN counts in BALF during 8 to 24 h after LPS i.t. TLR4 deficiency, however, prevented LPS-induced PMN infiltration in the lungs; whereas, IL-1RI and caspase-1 deficiency did not affect the LPS-induced PMN pulmonary infiltration at 8 h after LPS (early phase), but exhibited a suppressive effect on the PMN infiltration from 16 h to 24 h after LPS (late phase) (Fig. 5A). To determine a direct effect of pyroptotic AM on PMN chemotaxis, we collected AM from WT, IL-1RI^−/−^, and caspase-1^−/−^ mice, respectively, and treated the AM with LPS/ATP *in vitro* for 24 h. Typically, LPS/ATP treatments for 24 h cause pyroptosis in ~27% of WT AM, but only in ~5% of IL-1RI^−/−^ AM (Fig. 4A,B); and theoretically, pyroptosis does not occur in caspase-1^−/−^ AM. We then injected the LPS/ATP-treated WT, IL-1RI^−/−^, and caspase-1^−/−^ AM (1 × 10^5^ cells/ml saline/mouse) into mouse dorsal air pouch, respectively. At 8 and 16 h after macrophage injection, air pouch lavage fluid was collected for PMN counts. Figure 5B shows that WT AM induced PMN migration into the air pouch in a time-dependent manner. However, PMN migration into the air pouch significantly decreased in response to IL-1RI^−/−^ AM or caspase-1^−/−^ AM (Fig. 5B), suggesting a chemoattractant role of pyroptotic AM in inducing PMN tissue infiltration.

Cytokines IL-6, TNFα, and IL-1β in BALF were measured at 8 and 24 h after LPS i.t. (Fig. 5C,D,E). LPS induced the cytokines secretion in BALF in WT animals in a time-dependent manner. Noticeable, IL-1RI^−/−^ or caspase-1^−/−^ did not alter the BALF levels of IL-6 and TNFα at 8 h after LPS, but markedly attenuated the secretion of IL-6 and TNFα in BALF at 24 h after LPS. Similarly, IL-1RI^−/−^ did not influence IL-1β level in BALF at 8 h, but significantly decreased IL-1β secretion in response to LPS at 24 h. Caspase-1 deficiency prevented LPS-induced IL-1β secretion in BALF at both early and late time points.

Furthermore, lung histological analysis showed that IL-1RI^−/−^ and caspase-1^−/−^ markedly reduced neutrophil infiltration in capillary and extracapillary spaces as well as the degree of lung injury after LPS treatment as compared with that in WT mice (Fig. 5F). Taken together, these results suggest an important role of AM pyroptosis in augmenting lung inflammation.

## Discussion

**Figure 6:**
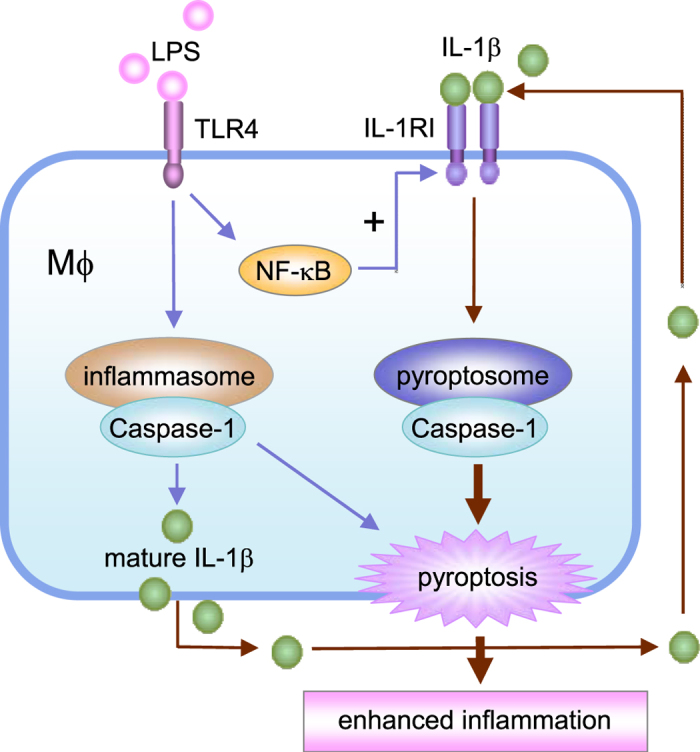
Hypothetic model for IL-1β–IL-1RI-mediatede AM Pyroptosis and Lung Inflammation in response to LPS. In AM LPS-TLR4 signaling not only activates Nlrp3 inflammasome activation and subsequent release of IL-1β, but also up-regulates IL-1RI cell surface expression through MyD88 and NF-κB dependent signaling. The upregulated IL-1RI, therefore, sensitizes AM to IL-1β and results in pyroptosome activation, which in turn leads to AM pyroptosis, a type of caspase-1-dependent inflammatory cell death, and subsequent exaggerated lung inflammation.

We observed in AM that LPS-TLR4 signaling not only activates Nlrp3 inflammasome activation and subsequent release of IL-1β, but also up-regulates IL-1RI expression on the AM surface through MyD88 and NF-κB dependent signaling. The upregulated IL-1RI, therefore, sensitizes AM to IL-1β and leads to pyroptosome formation and AM pyroptosis, a type of caspase-1-dependent inflammatory cell death, and subsequent augmented lung inflammation (Fig. 6). The present study demonstrates a feedback mechanism underlying LPS-induced innate immunity; that is, a secondary upregulation of IL-1β-IL-1RI signaling is responsible for AM pyroptosis and augmented lung injury in response to LPS.

The role of TLR4 signaling in regulating IL-1RI expression was demonstrated using TLR4^−/−^ mice. We showed that intratracheal injection of LPS into WT mice caused up-regulation of IL-1RI in AM, whereas the IL-1RI up-regulation was impaired in the TLR4^−/−^ mice. We also showed that MyD88 mediated the TLR4-IL-1RI cross-talk, since in MyD88^−/−^ mice LPS failed to increase IL-1RI expression. The phylogenetically conserved innate immunity signaling pathways activated by TLRs in mammals can induce responses through NF-κB^21^. In our *in vitro* study, we observed a diminished expression of IL-1RI in AM when NF-κB was inhibited by SN50, which supports a role of NF-κB in mediating TLR4-induced IL-1RI upregulation.

In the current study, by using a gene knockout approach, we showed that the Nlrp3 inflammasome functions as a key machine in the AM that induces IL-1β processing in response to LPS. The Nlrp3 inflammasome-induced IL-1β processing is an important up-steam event for the consequent pyroptosome formation and pyroptosis. This is evident by the fact that in Nlrp3 deficient AM, LPS failed to induce caspase-1 cleavage, IL-1β secretion, pyroptosome formation, and pyroptosis in both *in vitro* and *in vivo* settings; whereas, Nlrp3 deficiency did not affect IL-1β-induced pyroptosome formation and AM pyroptosis. These findings suggest an important role of Nlrp3 inflammasome in promoting mature IL-1β secretion and subsequent IL-1β-IL-1RI signaling activation rather than directly inducing AM pyroptosis.

Indeed, two distinct mechanisms of caspase-1 activation were identified after LPS stimulation of macrophages. An early detectable caspase-1 cleavage was observed starting at 3 h after LPS treatment, and this increased over time (Fig. 2A). This early activation of caspase-1 was prevented by Nlrp3 deficiency, indicating that this is an Nlrp3 inflammasome-dependent event. This Nlrp3 inflammasome-mediated caspase-1 activation resulted in increase in IL-1β release as shown in Fig. 2. Following ASC foci formation, we observed a late phase activation of caspase-1 at 16 h after LPS treatment (Fig. 4). This late caspase-1 activation was IL-1β-IL-1RI signaling dependent, since IL-1RI knockout diminished this LPS-induced caspase-1 activation, and IL-1β directly induced the activation of caspase-1 in both WT and Nlrp3 knockout AM, suggesting a pathway that depends on IL-1β and its receptor. Collectively, the data suggest that Nlrp3 inflammasome-activated caspase-1is required for IL-1β procession and release; and the released IL-1β acting through IL-1RI activates pyroptosomes, which further promote caspase-1 activation and subsequent pyroptosis.

Pyroptosis is a regulated form of inflammatory cell death^17^. Previous studies showed that pyroptosis can be triggered by members of the pattern recognition receptor (PRR) family, which includes TLRs and NOD-like receptors^17^. In this study, we demonstrated that LPS-induced AM pyroptosis is mainly mediated by a secondary signal, which is composed of upregulated IL-1β-IL-1RI loop. Our *in vivo* and *in vitro* studies show that associated with induction of AM pyroptosis, AM-originated inflammation, including PMN infiltration in the lungs and cytokines IL-6, TNFα, and IL-1β secretion, is augmented. By contrast, blocking LPS-induced AM pyroptosis by deletion of IL-1RI or caspase-1 significantly decreased the manifestation of inflammation and improved the lung histology (Fig. 5). The air pouch study showing a direct role of pyroptotic AM in inducing PMN migration further supports the influence of AM pyroptosis on the development of lung inflammation.

In summary, the present study demonstrates a novel function of upregulated IL-1β and IL-1RI signaling in mediating LPS-induced AM pyroptosis and subsequent enhanced lung inflammation. Since Nlrp3 inflammasome controls IL-1β processing and secretion, and pyroptosome formation (which is a down-stream event of IL-1β-IL-1RI signaling) leads to AM pyroptosis, the findings from the current study suggest that targeting multiple molecules may represent a therapeutic strategy for controlling inflammation during bacterial infection.

## Methods

### Mouse model of intratracheal injection of LPS or IL-1β

Male C57BL/6 wild type (WT) mice and IL-1RI knockout (IL-1RI^−/−^) mice were purchased from the Jackson Laboratory (Bar Harbor, ME). TLR4 knockout (TLR4^−/−^) mice and Nlrp3 knockout (Nlrp3^−/−^) mice were bred in Dr. Billiar’s lab at the University of Pittsburgh; all mice used are on a C57BL/6 background. All experimental protocols involving animals were performed in accordance with the recommendations in the Guide for the Care and Use of Laboratory Animals of the National Institutes of Health (Bethesda, MD, USA). All the animal experimental protocols were reviewed and approved by the Institutional Animal Care and Use Committee of VA Pittsburgh Healthcare System and University of Pittsburgh. All efforts were made to minimize suffering. Mice were 12–14 weeks of age at the time of experiments and were maintained on standard rodent chow and water *ad libitum*. The mice were not fasted before experiments. Animals were anesthetized with 50 mg/kg of ketamine and 5 mg/kg of xylazine via intraperitoneal (i.p.) administration. LPS (1 mg/kg B.W.; Escherichia coli, 0111:B4, 1 μg/ml, Sigma Aldrich, St. Louis, MO) or IL-1β (5 μg/kg B.W.; R&D Systems, Minneapolis, MN) in 0.2 ml of saline was injected intratracheally (i.t.) into the mice using a Micro Sprayer Syringe (PennCentury, Wyndmoor, PA). At various time points after LPS i.t. (0 to 24 h), either bronchoalveolar lavage (BAL) was performed and BAL fluid (BALF) was collected or lung tissue was harvested for experimental analysis.

### AM isolation

BAL was performed as previously described^22^. The immunomagnetic separation system (BD Biosciences Pharmingen, San Diego, CA) was used to isolate AM from BAL fluid. Magnetic nanoparticle-conjugated antibodies (anti-mouse Gr-1, anti-CD4, anti-CD8, and anti-CD45R/B220 antibodies; BD Biosciences Pharmingen, San Diego, CA) were chosen to label and remove PMN and lymphocytes. The resulting cells consisted of >98% macrophages, and cell viability was >95%.

### *In vitro* treatment of AM

AM isolated from BAL fluid were stimulated with LPS (Escherichia coli, 0111:B4, 1 μg/ml, Sigma Aldrich, St. Louis, MO) and 5 mM ATP (Sigma Aldrich, St. Louis, MO) for 0–24 h. In some experiments, AM were treated with IL-1β (5 ng/ml; R&D Systems, Minneapolis, MN) for 0–24 h. AM and supernatants were then collected for further analysis.

### Immunofluorescence confocal microscopy

AM were fixed with 4% paraformaldehyde for 20 min. After washing with PBS, the cells were permeabilized with 0.1% Triton X-100 in PBS for 10 min at room temperature, followed by blocking with 3% bovine serum albumin in PBST (PBS with 0.1% Tween-20) for 2 h at room temperature to reduce non-specific staining. The cells were then incubated with rabbit polyclonal anti-ASC Ab (Santa Cruz Biotechnology) at 4 °C overnight. After washing with PBS, the cells were incubated with Alexa Fluor 555-conjugated donkey anti-rabbit IgG (Abcam) for 1 h at room temperature. Hoechst 33258 (Sigma) was used to stain nuclei. The cells were then washed with PBS, followed by confocal microscopy. For visualize AM pyroptosis, the cells were incubated with Alexa Fluor 488-labeled caspase-1 FLICA at 37 °C for 1 h. After fixation with 4% paraformaldehyde, cells were stained with TMR red-labeled *In-Situ* Cell Death Detection reagent (Roche Applied Science, Indianapolis, IN) following the manufacturer’s instructions. The cells were then analyzed by confocal microscopy.

### Flow cytometry analysis of cell pyroptosis and IL-1RI expression

Two-color flow cytometry was used to detect cell pyroptosis. AM were incubated with Alexa Fluor 488-labeled caspase-1 FLICA at 37 °C for 1 h. After fixation with 4% paraformaldehyde, cells were stained with TMR red-labeled *In-Situ* Cell Death Detection reagent (Roche Applied Science, Indianapolis, IN) following the manufacturer’s instructions. The cells were then analyzed by flow cytometry. Background and auto-fluorescence were determined by a control antibody with the same isotype staining. Acquisition was performed on 10,000 events using a FACScalibur cytometer (BD Biosciences, San Jose, CA), CellQuestPro (BD Biosciences) and FlowJo-V10 software (Tree Star, Ashland, OR). The double-stained cells were considered to be pyroptotic cells, and the rate of pyroptotic cell was calculated as (pyroptotic cells/total cells) × 100%. For analysis of AM surface expression of IL-1RI, AM were stained with PE-tagged anti-IL-1RI antibody (R&D Systems, Minneapolis, MN), and flowcytometry was performed.

### Western Blot Analysis

Aliquots of AM lysates were separated on a 10% SDS-PAGE under non-reducing condition. Equivalent loading of the gel was determined by quantitation of protein as well as by re-probing the membranes for actin detection. Separated proteins were electroblotted onto PVDF membrane and blocked for 1 h at room temperature with Tris-buffered saline containing 1% BSA. The membranes were then probed with primary antibody (polyclonal anti-IL-1RI antibody purchased from Santa Cruz Biotechnology, Santa Cruz, CA) at room temperature for 1 h. After washing, primary antibodies associated with the membranes were detected on autoradiographic film by horseradish peroxidase-conjugated secondary antibodies and the ECL plus chemiluminescent system (Amersham, Arlington Heights, IL) according to the manufacturer’s instructions. Blots were quantitated using Scion Image software (Scion Corp., Frederick, MD) and normalized to actin. Caspase-1 cleavage in the AM was measured by detecting its p20 fragment in Western blot using rabbit polyclonal anti-mouse caspase-1 p20 (Santa Cruz Biotechnologies, CA).

### Measurement of cytokines

IL-1β in BALF and cell culture media was measured using ELISA Ready-Set-Go kit for mouse IL-1β (eBioscience, San Diego, CA) following the manufacturer’s instructions.

### *In vivo* air pouch animal model

Mouse air pouches were prepared as described^23^. In brief, WT mice were anesthetized, followed by injection of 5 ml sterile air under the dorsal skin. On day 3, the resultant space was again injected with 3 ml sterile air. On day 5, pyroptotic AM (1 × 10^5^ cells/ml saline/mouse) were injected into the air pouch. At 0, 8, and 16 h after macrophage injection, air pouch lavage fluid was collected for PMN counts.

### Lung histology scoring

Lung tissue was evaluated and graded blindly by applying an arbitrary grading scale ranging from 0 to 8 (0 = no changes, 1 = minimal lesions affecting 1–5% of the area, 2 = lesions affecting 6–10% of the area, 3 = changes affecting 11–15% of the area, 4 = changes affecting 16–20% of the area, 5 = changes affecting 21–25%, 6 = changes affecting 26–30%, 7 = severe tissue changes affecting 31–40, and 8 = tissue changes affecting >40% of the area). The following affected pulmonary parameters were evaluated: alveolar edema, alveolar hemorrhage, alveolar PMN infiltrates, interstitial cellular infiltrates and edema, perivascular cellular infiltrates and edema, and alveolar epithelial necrosis.

### Data presentation and statistical analysis

The data are presented as mean ± SEM of the indicated number of experiments. Statistical significance among group means was assessed by ANOVA. Student Neuman- Keuls *post-hoc* test was performed. Differences were considered significant at p < 0.05.

## Additional Information

**How to cite this article**: He, X. *et al*. TLR4-Upregulated IL-1ß and IL-1RI Promote Alveolar Macrophage Pyroptosis and Lung Inflammation through an Autocrine Mechanism. *Sci. Rep.* **6**, 31663; doi: 10.1038/srep31663 (2016).
